# Novel Therapeutic Strategies for Dyslipidemia: First Report of Inclisiran Therapy in a Kidney Transplanted Patient

**DOI:** 10.3389/ti.2023.11104

**Published:** 2023-01-26

**Authors:** Lars Ueberdiek, Ulrich Jehn, Hermann Pavenstädt, Katrin Gebauer, Stefan Reuter

**Affiliations:** ^1^ Department of Medicine D, Transplant Nephrology, University Clinics Muenster, Muenster, Germany; ^2^ Department of Cardiology I — Coronary and Peripheral Vascular Disease, Heart Failure, University Clinics Muenster, Muenster, Germany

**Keywords:** kidney transplantation, renal transplantation, dyslipidemia, inclisiran, LDL

Dear Editors,

Kidney transplant recipients are high-risk cardiovascular patients and cardiovascular events are the most common cause of death after kidney transplantation [1]. Management of cardiovascular risk factors, which includes adequate lowering of LDL cholesterol (LDLC) to the recommended levels, is difficult to achieve after renal transplantation or is not implemented consistently often enough [2]. This is partly because immunosuppressive therapies such as tacrolimus, prednisolone, or everolimus themselves have adverse effects on lipid levels and partly because there are incompatibilities and interactions between statins and immunosuppressive drugs i.e., ciclosporin A that limit adequate statin therapy and ezetimibe administration [3, 4].

Therefore, novel and highly efficient therapies such as inclisiran (SmPC Leqvio, Novartis, Germany) may contribute to better LDLC management in this patient population. Inclisiran is a small interference-RNA against protein convertase subtilisin/kexin type 9 (PCSK9), preventing LDL receptor degradation [5]. It is injected subcutaneously at month 0 and 3 and every 6 months thereafter and results in ∼50% LDLC reduction [6]. Inclisiran was first approved in the European Union in December 2020 for the treatment of primary hypercholesterolemia or mixed dyslipidemia in combination with a statin or other lipid-lowering therapies in patients who do not achieve LDLC goals with the maximum tolerable statin dose, or alone or in combination with other lipid-lowering therapies in patients with statin intolerance or for whom a statin is contraindicated.

To our knowledge, there is no data about the use of inclisiran in kidney transplant recipients yet. Therefore, we present for the first time a case of a patient treated with inclisiran after renal transplantation.

Our 79-year-old male patient received a deceased donor kidney transplant 12 years prior to the first inclisiran administration. End-stage renal disease was caused by right-sided nephrectomy due to renal cell carcinoma and unspecified nephrosclerosis of the left kidney. The immunosuppressive regimen at the time reported consisted of everolimus and prednisolone, due to a history of CMV disease. Serum creatinine was 2.44 mg/dL with an estimated GFR of 24 mL/min/m^2^ (CKD4A2T, CKD EPI). The patient has a distinct cardiovascular risk profile. In addition to male sex and older age, he suffers from metabolic syndrome (mixed dyslipidemia, arterial hypertension, post-transplant diabetes mellitus, BMI of 25 kg/m^2^) with hyperuricemia and has a history of smoking (approximately 13 pack years). This has led to progressive peripheral artery disease (Fontaine IIB) and coronary artery disease.

Serum lipids were inadequately controlled during therapy with atorvastatin 80 mg and ezetimibe 10 mg daily (total cholesterol 5.18 mmol/L, LDLC 2.46 mmol/L, HDLC 2.12 mmol/L and triglycerides 1.79 mmol/L). For our very high-risk patient, the 2019 ESC/EAS guidelines on the treatment of dyslipidaemia recommend a target LDLC of < 1.4 mmol/L and an LDLC reduction >50% from baseline values [7]. Therapeutic options were discussed with the patient and the patient opted for inclisiran therapy for optimal therapy adherence.

Inclisiran (284 mg s.c.) was administered at 0 and 3 months and then every 6 months while continuing atorvastatin and ezetimibe. LDLC was significantly lowered to 1.03, 1.14, and 1.32 mmol/L after 6, 9 and 12 months, respectively (Figure 1).

**FIGURE 1:**
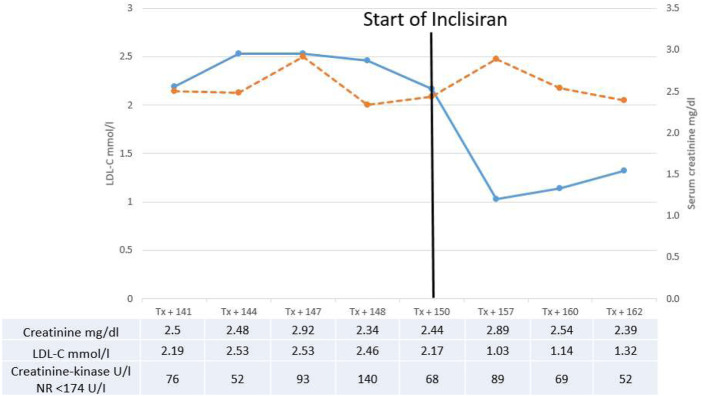
Serum-creatinine (dotted line) and LDL-cholesterol (line) before and after introduction of inclisiran therapy. Values of creatinine, LDL-cholesterol and creatinine-kinase are shown in the table. NR, normal range.

During the 1-year follow-up, renal function was stable after 12 months (serum creatinine 2.39 mg/dL, eGFR 25 mL/min/m^2^; Figure 1). We did not observe relevant side effects, or increase in proteinuria, creatinine-kinase or change in everolimus level.

The case presented demonstrates that inclisiran can be safely and conveniently administered with a profound effect on LDLC levels after renal transplantation. Further research needs to be conducted to demonstrate efficacy on cardiovascular death in transplanted patients.

## Data Availability

The data analyzed in this study is subject to the following licenses/restrictions: clinical data. Requests to access these datasets should be directed to the corresponding author.
