# Corrigendum: Novel Therapeutic Strategies for Dyslipidemia: First Report of Inclisiran Therapy in a Kidney Transplanted Patient

**DOI:** 10.3389/ti.2023.11313

**Published:** 2023-03-23

**Authors:** Lars Ueberdiek, Ulrich Jehn, Hermann Pavenstädt, Katrin Gebauer, Stefan Reuter

**Affiliations:** ^1^ Department of Medicine D, Transplant Nephrology, University Clinics Muenster, Muenster, Germany; ^2^ Department of Cardiology I—Coronary and Peripheral Vascular Disease, Heart Failure, University Clinics Muenster, Muenster, Germany

**Keywords:** kidney transplantation, renal transplantation, dyslipidemia, inclisiran, LDL

In the original article, there was an error. The injection type listed for the administration of Inclisiran was incorrect.

A correction has been made to paragraph 6:

“Inclisiran (284 mg i.m.) was administered at 0 and 3 months and then every 6 months while continuing atorvastatin and ezetimib.”

Has been updated to:

“Inclisiran (284 mg s.c.) was administered at 0 and 3 months and then every 6 months while continuing atorvastatin and ezetimibe.”

The authors apologize for this error and state that this does not change the scientific conclusions of the article in any way. The original article has been updated.

